# Optical Scattering Characteristics of Three-Dimensional Targets Based on Transfer Learning PINNs

**DOI:** 10.3390/mi17090991

**Published:** 2026-08-22

**Authors:** Renxian Li, Jiaze Huang, Huan Tang, Chenxu Zhou, Fengbei Liu, Zhe Wang

**Affiliations:** School of Physics, Xidian University, Xi’an 710071, China

**Keywords:** physics-informed neural networks, electromagnetic scattering, discrete maxwell operators, transfer learning

## Abstract

Physics-Informed Neural Networks (PINNs) continue to suffer from limited training efficiency in predicting electromagnetic scattering from three-dimensional (3D) targets, resulting in particularly high retraining costs when target geometries or incident fields vary. To address this issue, we propose a transferable PINN framework constrained by discrete Maxwell operators. By embedding finite-difference electromagnetic operators into the loss function, the predicted fields directly satisfy the discrete Maxwell’s equations on the computational grid, thereby enhancing physical consistency and improving training stability. Furthermore, a layer-freezing strategy is employed to preserve the generalized wave priors learned from the source scenario. Consequently, training under novel geometries or incident conditions no longer relies on full-network retraining; instead, rapid prediction is achieved by fine-tuning a minimal set of task-specific parameters. Numerical results demonstrate that the proposed method significantly reduces the number of iterations and retraining costs while maintaining prediction accuracy, making it an effective tool for rapid electromagnetic simulations and optical device design.

## 1. Introduction

In the fields of micro/nano-optics and computational electromagnetics, the accurate solution of three-dimensional (3D) electromagnetic scattering is a significant challenge encompassing both theoretical and practical considerations [1,2]. Due to the short wavelength of light, electromagnetic fields in complex 3D micro/nanostructures often exhibit rapid spatial oscillations, strong local enhancements, and multiple scattering, which impose high demands on numerical modeling and computational efficiency [3,4]. Therefore, developing efficient and accurate 3D electromagnetic scattering solution methods plays a crucial supporting role in the design of cutting-edge optical devices, such as metasurface lenses, optical integrated devices, and micro- and nano-optical field manipulation [5,6].

To address the challenge of accurately solving 3D electromagnetic scattering, traditional numerical methods such as the finite-difference frequency-domain (FDFD), finite-difference time-domain (FDTD), and finite element method (FEM) have been widely applied in modeling and simulation [7,8]. Based on the strict discrete solution of Maxwell’s equations, these methods possess clear physical significance and high numerical precision. They have achieved significant progress in areas including the solution of single-frequency steady-state fields in 3D complex media [9,10], the design of high-power microwave and terahertz devices [11,12], as well as the stable computation of large-scale, high-contrast dielectric systems [13,14], thereby providing a reliable theoretical foundation and effective computational tools for the precise analysis of complex 3D electromagnetic scattering problems.

To meet the design requirements of complex micro/nano-optical devices, traditional numerical electromagnetic methods, despite their high accuracy and clear physical foundations, incur high computational costs for multi-scenario predictions and inverse designs [15,16]. When the target geometry, material distribution, or incident conditions change, traditional methods often require rebuilding discrete models and repeating the solution process, making it difficult to reuse existing field response information fully [17]. Furthermore, in optimization tasks such as metasurface manipulation and low-scattering structure design, the number of simulations can be massive, further exacerbating the computational burden. Consequently, developing rapid solution methods that combine Maxwell physical constraints, field response learning capabilities, and cross-scenario transferability is a vital approach to enhancing the design efficiency of complex micro/nano-optical devices [18,19,20].

At present, data-driven deep learning methods have been widely adopted in computational electromagnetics and micro- and nano-optics [21]. These methods typically construct a mapping between the permittivity distribution and the electromagnetic scattering field from numerical simulation data, then employ neural networks to approximate this mapping [22,23]. Once trained, the network models can significantly reduce forward prediction costs and provide rapid inversion models for the inverse design of structural parameters under optical response constraints [24]. Currently, data-driven deep learning methods have demonstrated high efficiency in electromagnetic forward prediction and inverse design [25]. Parameterized neural networks can achieve multi-scenario predictions within a given range by incorporating geometry, material, or incident conditions. Additionally, inverse and tandem neural networks have been utilized for structural parameter inversion [26,27]. However, such methods usually rely on large-scale annotated data, and their predictions lack explicit physical constraints, limiting their generalization and reliability in complex scattering scenarios [28].

To overcome the reliance of purely data-driven methods on large-scale annotated data and their lack of physical constraints, Physics-Informed Neural Networks (PINNs) are introduced into the fields of computational electromagnetics and micro/nano-optics [29]. By embedding Maxwell’s equations and boundary conditions into the loss function, PINNs constrain the network’s training with electromagnetic physical laws, thereby providing a solution paradigm for complex electromagnetic scattering problems that balances learning capability and physical consistency [19,30]. It achieves forward prediction of electromagnetic fields under unlabeled or sparsely labeled conditions and enhances model robustness to sparse observations and noise interference in parameter inversion tasks [31,32]. Furthermore, unlike traditional numerical methods, a trained PINN can directly output the electromagnetic field distribution through network forward propagation. This significantly accelerates the prediction process, taking only a few seconds.

However, when the target geometric structures or incident beams change, PINNs typically still require retraining or extensive parameter adjustments, which limits their cross-scenario solution efficiency [33]. To solve this issue, transfer learning strategies integrated into electromagnetic PINNs to reuse the network weights or field-response features learned in source scenarios [34]. Recently, methods such as global fine-tuning, meta-learning, residual transfer, and operator learning have improved the model’s adaptability to novel scenarios to varying degrees [35,36,37,38]. Nevertheless, existing transfer learning methods primarily focus on reusing network parameters or features, often still necessitating substantial parameter adjustments in new scenarios, resulting in high adaptation costs [39]. Simultaneously, these methods do not alter how PINNs enforce physical constraints through continuous automatic-differentiation residuals. Thus, they may still encounter training instability in 3D high-frequency scattering problems.

Therefore, this paper proposes a transferable PINN framework constrained by discrete Maxwell operators to address insufficient training stability and high cross-scenario retraining costs in 3D high-frequency electromagnetic scattering. This method embeds the finite-difference Maxwell operators into the loss function to strengthen electromagnetic physical constraints on the discrete computational grid. Concurrently, it incorporates a layer-freezing strategy to reuse learned scattering priors, enabling rapid predictions across different geometric structures and beam conditions. The remainder of this paper is organized as follows: Section 2 presents the governing equations for 3D electromagnetic scattering and the proposed transferable PINN framework. Section 3 conducts numerical validation using the FDFD method as a benchmark, analyzing the prediction accuracy, training efficiency, and cross-scenario transferability of the transferable PINN method. Section 4 concludes the paper and anticipates its applications in rapid electromagnetic simulation and the inverse design of photonic devices.

## 2. Methods

By focusing on the governing equations for 3D electromagnetic scattering and the layer-freezing transfer strategy, this method constructs a PINN-based electromagnetic scattering solution framework. It serves as the theoretical foundation for efficiently predicting field distributions in complex dielectric targets in this study.

### 2.1. Governing Equations

Assuming the electromagnetic field varies harmonically with time (containing the $e^{+i\omega t}\;$ factor, where ω is the angular frequency), the propagation of electromagnetic waves in 3D space within a source-free, linear, and isotropic medium is governed by the following frequency-domain Maxwell’s equations [40,41]:

(1) ∇×E=−iωμ(r)H,

(2) ∇×H=iωϵ(r)E,

Here, E and H are the spatial total electric and magnetic field vectors, respectively. $\mu (r)\;=\;\mu_{0}\mu_{r}(r)$ and $\epsilon (r)=\epsilon_{0}\epsilon_{r}(r)$ represent the space-dependent permeability and permittivity, respectively, where r=(x,y,z) represents the spatial position vector in the 3D domain.

For the targets studied in this paper, the relative permeability is globally constant at $\mu_{r}=1$, where $\mu_{0}$ is the vacuum permeability and $\epsilon_{r}(r)$ is the vacuum permittivity. The relative permittivity $\epsilon_{r}(r)$ exhibits a step-discontinuous distribution in space: $\epsilon_{r}(r)=2.0$ inside the target, and $\epsilon_{r}(r)=1.0$ in the background vacuum in this paper.

To formulate the scattering problem, the total electromagnetic field is separated into two parts: an analytically known incident field ($E_{inc}$, $H_{inc}$) and an unknown scattered field to be solved ($E_{sca}$, $H_{sca}$) [42,43]:

(3) $$E=E_{inc}+E_{sca},H=H_{inc}+H_{sca}.$$

The incident field strictly satisfies the governing equations of free space in a homogeneous background medium (with relative permittivity $\epsilon_{bg}$ and relative permeability $\mu_{bg}$) in the absence of any scatterers:

(4) $$\nabla \times E_{inc}=-i\omega \mu_{0}\mu_{bg}H_{inc},$$

(5) $$\nabla \times H_{inc}=i\omega \epsilon_{0}\epsilon_{bg}E_{inc}.$$

Substituting Equations (3)–(5) into Equations (1) and (2), and eliminating the incident field using the linearity of the operators, a system of governing equations can be derived with only the scattered field ($E_{sca}$, $H_{sca}$) as the unknown variables:

(6) $$\nabla \times E_{sca}=-i\omega \mu (r)H_{sca}-i\omega (\mu (r)-\mu_{0}\mu_{bg})H_{inc},$$

(7) $$\nabla \times H_{sca}=i\omega \epsilon (r)E_{sca}+i\omega (\epsilon (r)-\epsilon_{0}\epsilon_{bg})E_{inc}.$$

To ensure that the finite computational domain can represent an infinitely large, boundless physical space, it is necessary to eliminate non-physical reflections caused by grid truncation. To this end, this paper introduces the stretched-coordinate perfectly matched layer (PML) [44,45]. Through analytical continuation, it modifies the conventional spatial derivative $\frac{\partial }{\partial w}$ in the Cartesian coordinate system to $\frac{1}{s_{w}}\frac{\partial }{\partial w}$ (where w∈{x,y,z}). Here, $s_{w}$ is the complex stretching factor in that direction, defined as:

(8) $$s_{w}\left(w\right)=1+i\frac{\sigma_{w}\left(w\right)}{\omega \epsilon_{0}},$$

where $\sigma_{w}(w)$ is the conductivity attenuation profile function. Inside the physical computational domain, $\sigma_{w}\equiv 0$, yielding $s_{w}=1$, and the equations degenerate into Maxwell’s equations in conventional space. Whereas within the boundary PML region, $\sigma_{w}\left(w\right)$ increases polynomially along the normal vector, causing the radiating waves entering this region to exhibit reflectionless exponential decay. In discrete computations, this paper defines a dimensionless complex boundary tensor parameter $\Phi_{w}=s_{w}(w)$ as the core variable for constructing the absorbing boundary. It is crucial to note that $\Phi_{x}$ is a spatially dependent parameter rather than a global constant: it equals 1 inside the physical domain and becomes a complex value exclusively within its corresponding boundary region.

To construct physical constraints, this paper maps the modified equations onto a 3D staggered Yee grid [46]. In this grid, the electric field E and magnetic field H are stagger-distributed by half-step, which naturally fits Maxwell’s curl equations and implicitly satisfies divergence conservation [47,48]. Consequently, the spatial partial derivatives containing the PML tensor are transformed into first order forward and backward sparse difference matrix operators ($D_{w}^{e}$ and $D_{w}^{h}$).

Therefore, instead of adding complex boundary penalty terms to the loss function, this paper embeds the complex tensor $\Phi_{w}$ into the boundaries of the difference matrices. Taking the forward difference matrix $D_{x}^{e}$ in the x-direction as an example, the absorbing parameter $\Phi_{x}$ is mapped to the boundary truncation elements of this coefficient matrix:

(9) $$D_{x}^{e}=\frac{1}{\Delta x}\left[\begin{array}{ccccc} -1 & 1 & 0 & \ldots & 0\\ 0 & -1 & 1 & \ldots & 0\\ \vdots & \vdots & \ddots & \ddots & \vdots \\ 0 & 0 & \ldots & -1 & 1\\ \Phi_{x} & 0 & \ldots & 0 & -1 \end{array}\right].$$

Simultaneously, the corresponding backward difference matrix $D_{x}^{h}$ follows the transposition rule $D_{x}^{h}=-(D_{x}^{e}{)}^{T}$, and its specific form also incorporates the boundary absorbing parameter:

(10) $$D_{x}^{h}=\frac{1}{\Delta x}\left[\begin{array}{ccccc} 1 & 0 & \ldots & 0 & -\Phi_{x}\\ -1 & 1 & \ldots & 0 & 0\\ \vdots & \ddots & \ddots & \vdots & \vdots \\ 0 & \ldots & -1 & 1 & 0\\ 0 & \ldots & 0 & -1 & 1 \end{array}\right].$$

As shown in Equations (9) and (10), the same boundary variable $\Phi_{x}$ is utilized in both forward ($D_{x}^{e}$) and backward ($D_{x}^{h}$) difference matrices because they operate on the identical physical x-boundary layers for E-field and H-field, respectively. Similarly, the forward and backward difference matrices for the y-direction and z-direction are constructed analogously, utilizing their strictly independent anisotropic boundary parameters $\Phi_{y}$ and $\Phi_{z}$ to absorb waves propagating along those respective axes.

With the aid of the aforementioned sparse difference operators incorporating PML boundaries, the curl operation (∇×) in continuous space is transformed into global block matrices $C_{E}$ and $C_{H}$ [49]:

(11) $$C_{E}=\left[\begin{array}{ccc} 0 & -D_{z}^{e} & D_{y}^{e}\\ D_{z}^{e} & 0 & -D_{x}^{e}\\ -D_{y}^{e} & D_{x}^{e} & 0 \end{array}\right],C_{H}=\left[\begin{array}{ccc} 0 & -D_{z}^{h} & D_{y}^{h}\\ D_{z}^{h} & 0 & -D_{x}^{h}\\ -D_{y}^{h} & D_{x}^{h} & 0 \end{array}\right].$$

Thus, substituting Equation (11) into Equations (6) and (7) yields the discretized system of equations:

(12) $$C_{E}E_{sca}=-i\omega \mu H_{sca}-i\omega (\mu -\mu_{bg})H_{inc},$$

(13) $$C_{H}H_{sca}=i\omega \epsilon E_{sca}+i\omega (\epsilon -\epsilon_{bg})E_{inc}.$$

Since the global relative permeability constantly satisfies $\mu =\mu_{bg}$, Equation (12) can be simplified to:

(14) $$C_{E}E_{sca}=-i\omega \mu_{0}H_{sca}⟹H_{sca}=\frac{i}{\omega \mu_{0}}C_{E}E_{sca}.$$

Substituting Equation (14) into Equation (13) eliminates the scattered magnetic field component $H_{sca}$. After simplification, a discrete second-order decoupled vector wave equation system with the scattered electric field $E_{sca}$ as the sole unknown is obtained:

(15) $$(C_{H}C_{E}-\omega^{2}\mu_{0}\epsilon )E_{sca}=\omega^{2}\mu_{0}(\epsilon -\epsilon_{bg})E_{inc}.$$

To simplify the expression, Equation (15) can be abbreviated as a sparse matrix equation:

(16) $$A_{E}E_{sca}=B_{E},$$

where $A_{E}=C_{H}C_{E}-\omega^{2}\mu_{0}\epsilon$ is the physical operator integrating the double-curl matrix and the inhomogeneous response of the medium. The right-hand side term $B_{E}=\omega^{2}\mu_{0}(\epsilon -\epsilon_{bg})E_{inc}$ acts as the equivalent polarization current source exciting the global scattered field.

At this point, according to Equation (16), the physical accuracy of any scattered field $E_{sca}^{pred}$ predicted by PINNs can be evaluated by calculating its discrete algebraic residuals (R) [50]:

(17) $$R\left(E_{sca}^{pred}\right):=A_{E}E_{sca}^{pred}-B_{E}.$$

Furthermore, considering the magnitude imbalances often present in high-frequency electromagnetic fields at local enhancement regions or interference extrema, conventional mean squared error (MSE) metrics lead to the loss of critical phase features. Therefore, this paper introduces a normalization strategy based on the global norm of the equivalent source term, ultimately constructing the following 3D physical residual metric function (i.e., the PDE loss criterion subsequently used to drive the solution) [51]:

(18) $$L_{PDE}(E_{sca}^{pred})=\sum_{w\in \{x,y,z\}}\left(\frac{MSE(Re(R_{w}(E_{sca}^{pred})))}{\|Re(B_{E,w}){\|}_{2}^{2}}+\frac{MSE(Im(R_{w}(E_{sca}^{pred})))}{\|Im(B_{E,w}){\|}_{2}^{2}}\right),$$

(19) $$L_{total}={\lambda_{PDE}L}_{PDE}+{\lambda_{data}L}_{data}$$

where $L_{total}$ denotes the total objective function to be minimized during training, and $L_{data}$ represents the data loss. The parameters $\lambda_{PDE}$ and $\lambda_{data}$ are their respective weighting coefficients. Since no labeled data is utilized in this paper, we set $\lambda_{data}$ = 0 and $\lambda_{PDE}$ = 1. Therefore, $L_{total}$ is driven exclusively by the physical residuals. The detailed configuration is described below.

### 2.2. Architecture of the Physics-Informed Neural Network

To solve the discrete governing equations, this paper constructs a fully connected feedforward neural network (MLP, $N_{\Theta }$). The network comprises 4 hidden layers, each with 256 neurons. To enhance the fitting sensitivity to high-frequency fluctuating signals, the Sine function [52] is employed as the activation function for all hidden layers. The input dimension of the network is 3, corresponding to the Cartesian spatial coordinates (x,y,z) in the physical computational domain. The output dimension is 6, corresponding to the real and imaginary parts of the orthogonal components $E_{sca}^{x}$, $E_{sca}^{y}$, and $E_{sca}^{z}$ of the scattered electric field vector $E_{sca}$. Consequently, the network establishes a continuous mapping from the spatial input coordinates to the scattered field outputs, which is formulated as:

(20) $$N_{\Theta }(x,y,z)\mapsto [Re(E_{sca}^{x}),Im(E_{sca}^{x}),Re(E_{sca}^{y}),Im(E_{sca}^{y}),Re(E_{sca}^{z}),Im(E_{sca}^{z})].$$

The scattered magnetic field components ($H_{sca}$) are not directly predicted but strictly deduced via Faraday’s law from Equation (7), thereby ensuring electromagnetic consistency.

The network training relies entirely on spatial collocation points. These points consist solely of spatial coordinates (x,y,z) within the computational domain and contain no field labels or supervised data. Spatially, these collocation points are strictly fixed at the discrete nodes of a 3D staggered Yee grid, rather than being randomly sampled, to accommodate the finite-difference Maxwell operators used in the loss function. The neural network takes these coordinates as input and outputs predicted fields, which are then substituted into the discrete operators to compute physical residuals (Equation (17)). Furthermore, for all target structures examined in this study, the spatial distribution of these collocation points remains unchanged. Ultimately, the model is optimized by minimizing the global PDE loss function $L_{PDE}$ (Equation (18)), without relying on any external data.

### 2.3. Transfer Learning

After constructing a loss function constrained by Maxwell’s equations, the layer-freezing transfer strategy is introduced to overcome the cross-scenario transfer bottleneck faced by traditional paradigms [53,54].

Transfer learning relies on a baseline geometry model with robust physical deduction capabilities. In this paper, under a scenario involving a representative scatterer and a standard incident wave (e.g., a plane wave), the entire network is sufficiently trained using the total loss function derived above.

During this stage, the network parameters are initialized randomly and updated over hundreds of thousands of iterations, gradually embedding the divergence and curl laws of Maxwell’s equations into the neural network’s weight matrices. Through this process, the baseline model learns a near-essential physical law: how electromagnetic waves refract, diffract, and scatter in the presence of an arbitrary continuous dielectric gradient. This memory and learning of Maxwell’s equation operators lay the foundation for subsequent cross-scenario transfer.

To achieve efficient transfer, it is necessary to deeply understand the hierarchical structure of feature extraction within PINNs, as well as the feature coupling phenomenon exhibited by multi-layer neural networks when fitting PDEs [55,56].

1. Shallow Networks (Physical Perception Layers): The shallow weights adjacent to the input terminals primarily capture global, low-frequency universal physical laws (such as the basic mapping of wave operators). These features are highly universal across different electromagnetic scattering scenarios.

2. Deep Networks (Geometric Mapping Layers): The deep weights near the output terminals are primarily responsible for fitting high-frequency details, specific geometric structures, and local field intensities in the current scenario.

Assuming the parameter set of the pre-trained baseline geometric target is $\theta_{base}$, when encountering a brand-new physical scenario (such as an unseen complex-shaped target), we initialize the new network with these pre-trained weights (i.e., $\theta^{\prime }=\theta_{base}$) and hierarchically divide them into two parts:

(21) $$\theta^{\prime }=\left[\theta_{fro},\theta_{tun}\right],$$

where $\theta_{fro}$ (frozen parameters) contains the first hidden layers. During the transfer process, this portion of parameters is kept fixed and is excluded from gradient-based updates, while still participating in the forward propagation. $\theta_{tun}\;$(tunable parameters) encompasses the remaining deep hidden layers and the final linear output layer. These parameters serve as trainable variables to be fine-tuned using the loss function for the new scenario. This layer-freezing strategy substantially reduces the volume of trainable parameters. It smooths the loss landscape [57], allowing the model to rapidly converge to a high-precision physical field with only a few iterations when faced with new physical scenarios.

During the transfer stage, since this paper only needs to fit the local geometric changes introduced by the new target $d_{new}(r)$ (or a new incident wave $E_{inc}^{new}$), the optimization formula for the fine-tuning stage $\theta_{tun}^{*}$ is simplified to:

(22) $$\theta_{tun}^{*}=\underset{\theta_{tun}}{argmin}L\left(\theta_{fro},\theta_{tun};d_{new}\left(r\right),E_{inc}^{new}\right).$$

It is crucial to emphasize that the framework’s ability to evaluate complex targets does not stem from learning data mapping from geometric structures to scattered fields. Instead, for any new target, its spatial permittivity distribution is utilized to reconstruct the discrete Maxwell operators. During the fine-tuning stage (Equation (22)), the pre-trained baseline weights merely provide a physically relevant parameter initialization suitable for representing oscillating electromagnetic fields. Since the update of network parameters is driven entirely by minimizing the Maxwell equation residuals specific to the new target, the final predicted scattering field is strictly determined by the physical constraints of the new geometry. The complete step-by-step procedure for this transfer learning process is explicitly summarized in Algorithm 1.
**Algorithm 1.** TL-PINNs Pretraining and Layer-Freezing Fine-Tuning**INPUT:** Baseline permittivity ϵ(x,y,z), coordinates (x,y,z), baseline incident field $\;\;\;\;\;E_{inc}$ **OUTPUT:**$\;\mathrm{Predicted}\;\mathrm{scattered}\;\mathrm{field}\;E_{sca}^{pred}$ for the new target scenario 
    01: Randomly initialize baseline parameters θ
$\;\;\;\;02:\;\mathrm{Set}\;\mathrm{PDE}\;\mathrm{loss}\;L_{PDE}$$\;\mathrm{using}\;\mathrm{baseline}\;\mathrm{discrete}\;\mathrm{operators}\;A_{E}$$\;\mathrm{and}\;\mathrm{source}\;B_{E}$
    03: for t←1$\;\mathrm{to}\;T_{pre}$ **do**
    04:     Sample collocation points (x,y,z) from 3D Yee grid 
$\;\;\;\;05:\;\;\;\;\;\mathrm{Compute}\;L_{PDE}$ under baseline constraints 
    06:     Update θ via Adam and L-BFGS
    07: end for
$\;\;\;\;08:\;\mathrm{Save}\;\text{pre-trained}\;\mathrm{weights}\;\mathrm{as}\;\theta_{base}$
$\;\;\;\;09:\;\mathrm{Construct}\;\mathrm{target}\;\mathrm{problem}:\;\mathrm{update}\;\mathrm{permittivity}\;\mathrm{for}\;\mathrm{new}\;\mathrm{geometry}\;d_{new}(r)$ or set 
$\;\;\;\mathrm{new}\;\mathrm{incident}\;\mathrm{wave}\;E_{inc}^{new}$
$\;\;\;\;10:\;\mathrm{Transfer}\;\mathrm{weights}\;\mathrm{to}\;\mathrm{target}\;\mathrm{network}:\;\theta^{\prime }\leftarrow \theta_{base}$
$\;\;\;\;11:\;\mathrm{Partition}\;\mathrm{and}\;\mathrm{freeze}\;\mathrm{layers}:\theta^{\prime }=\left[\theta_{fro},\theta_{tun}\right]$$\;(\mathrm{freeze}\;\mathrm{shallow}\;\theta_{fro}$, tune deep
$\;\;\;\theta_{tun})$
$\;\;\;\;12:\;\mathrm{Set}\;\text{fine-tuning}\;\mathrm{loss}\;{L^{\prime }}_{PDE}$ under new target constraints 
    13: for t←1$\;\mathrm{to}\;T_{tra}$ **do**
    14:     Sample collocation points (x,y,z)
$\;\;\;\;15:\;\;\;\;\;\mathrm{Compute}\;{L^{\prime }}_{PDE}$ under new target constraints 
$\;\;\;\;16:\;\;\;\;\;\text{Fine-tune}\;\mathrm{deep}\;\mathrm{parameters}\;\theta_{tun}$ using target loss     17:end for
$\;\;\;\;18:\mathrm{Save}\;\text{fine-tuning}\;\mathrm{weights}\;\mathrm{as}\;\theta_{tuned}$
$\;\;\;\;19:\mathrm{Define}\;\mathrm{the}\;\mathrm{evaluation}\;\mathrm{coordinates}\;(x^{\prime },y^{\prime },z^{\prime })$ for the required spatial domain 
$\;\;\;\;20:\mathrm{Load}\;\mathrm{tuned}\;\mathrm{weights}\;\theta_{tuned}$ to target network 
$\;\;\;\;21:\mathrm{Compute}\;E_{sca}^{pred}$$\;\mathrm{at}\;(x^{\prime },y^{\prime },z^{\prime })$ via a forward pass of the network

TL-PINN Pretraining and Layer-Freezing Fine-Tuning. The algorithm outlines the complete transfer learning framework for predicting electromagnetic scattered fields. It details the initial pretraining of the network on a baseline scenario to extract universal wave priors, followed by a layer-freezing fine-tuning stage where only deep parameters ($\theta_{tun}$) are updated to adapt to novel geometric boundaries or incident waves while keeping the shallow layers ($\theta_{fro}$) frozen. Furthermore, it explicitly incorporates the final inference stage: after saving the fine-tuned weights ($\theta_{tuned}$), the target network performs rapid forward propagation to directly compute the predicted scattered field ($E_{sca}^{pred}$) at any desired evaluation coordinates $\left(x^{\prime },y^{\prime },z^{\prime }\right)$. Here, $T_{pre}$ and $T_{tra}$ denote the maximum number of iterations for the baseline model and the transfer model, respectively.

## 3. Numerical Calculations and Discussions

### 3.1. Experimental Setup and Evaluation Metrics

To systematically evaluate the proposed TL-PINN framework, this section outlines the physical parameters and geometry representation, training and optimization strategy, evaluation metrics, and hardware configuration used in the numerical simulations.

First, regarding the physical parameters and geometry representation, in all 3D high-frequency electromagnetic scattering simulations herein, the free-space incident wavelength is set to λ=0.532 μm (operating frequency f≈563.9 THz). The background medium is vacuum ($\epsilon_{r1}=1.0$), and the scattering targets possess a real relative permittivity of $\epsilon_{r2}=2.0$. To isolate and validate the convergence properties of the discrete Maxwell operators, the current study focuses exclusively on non-dispersive, lossless dielectric media.

Next, for modeling complex geometries (e.g., hemispheres, rounded cubes, and dual-sphere systems), the Signed Distance Function (SDF) [58] is employed to generate precise discrete dielectric masks across the computational grid.

It should be noted that these target geometries are not treated as explicit input parameters for the neural network. Instead, they are implicitly represented by the spatial distribution of permittivity across the computational domain, which is then directly incorporated into the discrete Maxwell operators [59].

Then, in terms of the training and optimization strategy. The partial differential equation (PDE) loss landscape for high-frequency optical scattering typically exhibits non-convexity and local oscillations [60]. A single optimizer struggles to balance initial descent speed and final convergence accuracy. Therefore, this experiment implements a customized two-stage hybrid training strategy:

Stage 1: In the early stage of training, the Adam optimizer with a stepwise dynamic learning-rate decay strategy is employed [61]. A higher learning rate is initially set to break through local optima and achieve a rapid decline in PDE loss. Subsequently, as training progresses, to avoid violent PDE oscillations and approach the optimal solution, the learning rate decays progressively, guiding the PDE to converge at the optimal point. Based on experience, this paper adopts four phases, changing the learning rate every 200,000 iterations, from 5 × 10^−3^ to 2.5 × 10^−3^, then to 1 × 10^−3^, and finally to 5 × 10^−4^.

Stage 2: When the first-order Adam optimizer enters a plateau, the loss curve is highly prone to oscillations, making it difficult to further fit the microscopic details of the physical field. At this point, the optimizer is switched to L-BFGS [62]. Utilizing its second-order Hessian matrix, L-BFGS can effectively suppress oscillations, guide the total loss function $L_{PDE}$ to break through bottlenecks, and converge to a high-precision level.

Subsequently, for the evaluation of metrics. Regarding model accuracy, this paper evaluates it using the difference between the scattered field predicted by PINNs ($E_{pred}$) and the benchmark field calculated by FDFD ($E_{ref}$). This paper selects absolute error and relative error as the evaluation metrics.

The absolute error is defined as:

(23) $${Error}_{abs}(r)=\left|E_{pred}\left(r\right)-E_{ref}\left(r\right)\right|.$$

The relative error is defined as:

(24) $${Error}_{rel}(r)=\frac{{Error}_{abs}(r)}{max(|E_{ref}(r)|)}.$$

The overall relative error reported herein is defined as the maximum of ${Error}_{rel}(r)$ across the entire computational domain. In this paper, we define a maximum relative error of less than 10% as the acceptable limit. Within this limit, both the PINN and TL-PINN models exhibit high prediction accuracy, yielding results that fully satisfy computational demands.

Finally, regarding the hardware configuration, all neural network models in this study were trained and evaluated on a workstation equipped with an NVIDIA RTX A6000 GPU (NVIDIA, Santa Clara, CA, USA).

### 3.2. Accuracy Analysis of the Baseline Geometric Target

To verify the accuracy of the proposed framework for solving 3D high-frequency electromagnetic problems, this paper first analyzes the baseline geometric target (a standard dielectric sphere) during the pre-training phase. We compare the PINN-predicted field with the results from traditional FDFD solvers.

**Figure 1:**
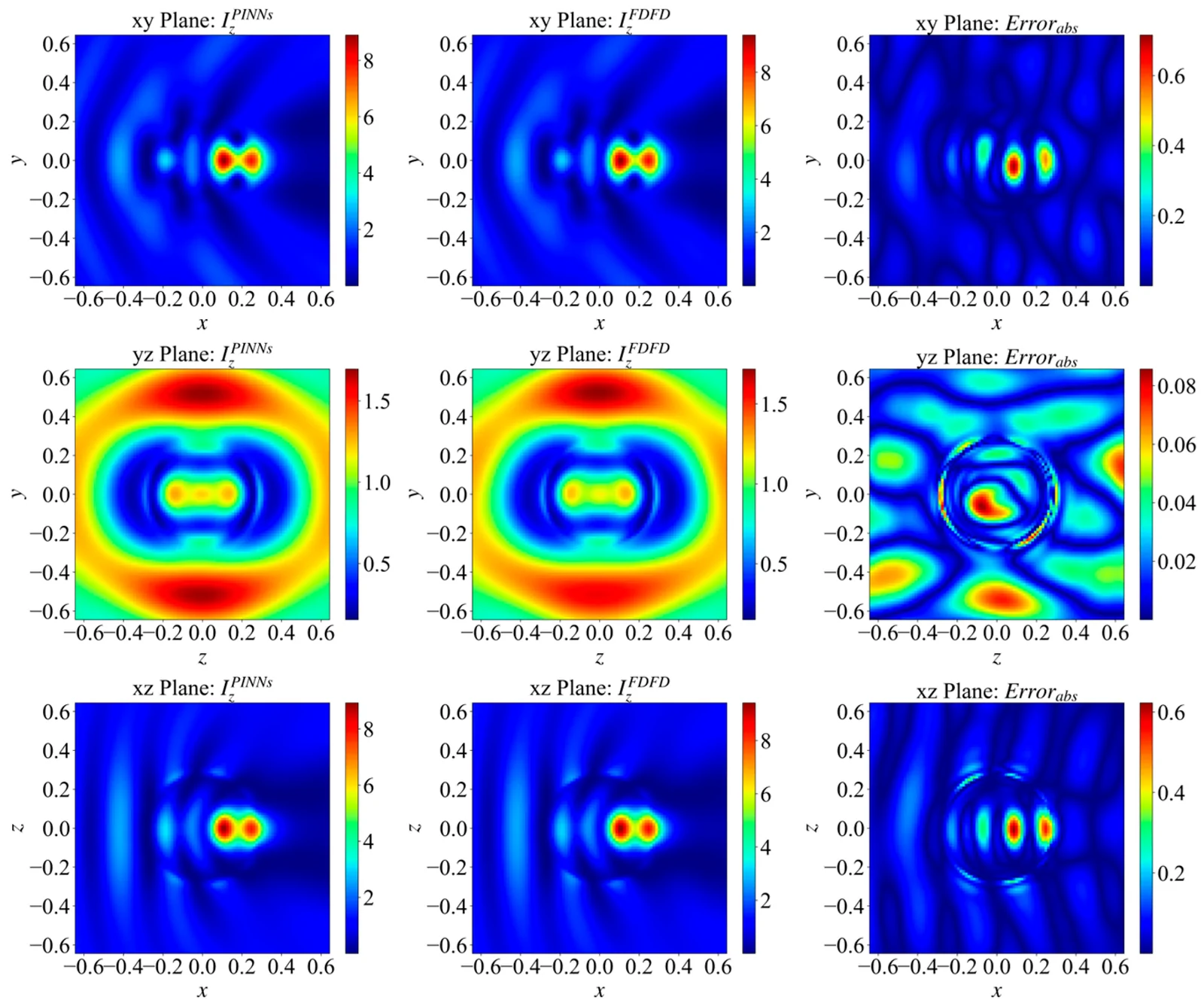
Electric field intensity distributions on the central cross-sections for the baseline dielectric sphere. The incident plane wave propagates along the x-direction with z-polarization. The subplots compare the predicted fields by PINNs (left column), the benchmark fields calculated by FDFD (middle column), and the corresponding spatial error distributions (right column) across the xy, yz, and xz planes.

The computational domain selected in this paper ranges from −0.798 μm (−1.5 λ) to 0.798 μm (1.5 λ), the chosen PML thickness is 0.1596 μm (0.3 λ), and the selected target radius is 0.266 μm (0.5 λ), with a grid size of 120×120×120. Figure 1 illustrates the intensity distribution of the electric field components on the central cross-sections. The incident wave is a plane wave, propagating along the x-direction and polarized in the z-direction. The figures show that the field predicted by PINNs (left) is highly consistent with the field calculated by FDFD (middle).

Observing the field intensity distribution in the xy plane, it is evident that the incident wave refracts and converges after entering the dielectric sphere. This process locally forms two distinct high-intensity focal regions. A comparison of the absolute error (${Error}_{abs}$) distributions reveals that PINNs accurately predict the spatial locations of these focal regions. However, significant error extrema occur precisely at the field-intensity peaks. This discrepancy can be attributed to the inherent “spectral bias” characteristic of neural networks [63,64]. Physically, the focal peaks exhibit rapid spatial variations and steep gradients, which correspond to high-frequency components in the spatial domain. Because PINNs tend to preferentially fit low-frequency, smooth components during gradient descent, the model underfits these high-frequency peaks in field intensity. Consequently, the predicted peak intensities fail to reach the true calculated maximums. Furthermore, the wavefield fringes dominated by interference and scattering at the periphery of the xy plane exhibit high morphological consistency with the FDFD benchmark.

In the error distribution maps of the xz and yz planes, errors are primarily concentrated at the interface between the dielectric sphere and the vacuum. This concentration exhibits clear annular error bands. The underlying physical cause of this phenomenon is that as electromagnetic waves cross different media, there is a spatial step discontinuity in the permittivity. This discontinuity requires the physical field (or its derivatives) to satisfy strict boundary conditions. However, PINNs are inherently continuously differentiable function approximators. Therefore, under a limited neuron capacity, it is difficult for them to characterize such discontinuous jumps in physical quantities accurately. To fit the true boundary responses, the network must generate sharp changes in gradient within an extremely narrow transition zone. This high-frequency gradient oscillation significantly increases the difficulty of convergence for the optimizer. Ultimately, this induces concentrated annular error distributions at the dielectric boundaries.

**Figure 2:**
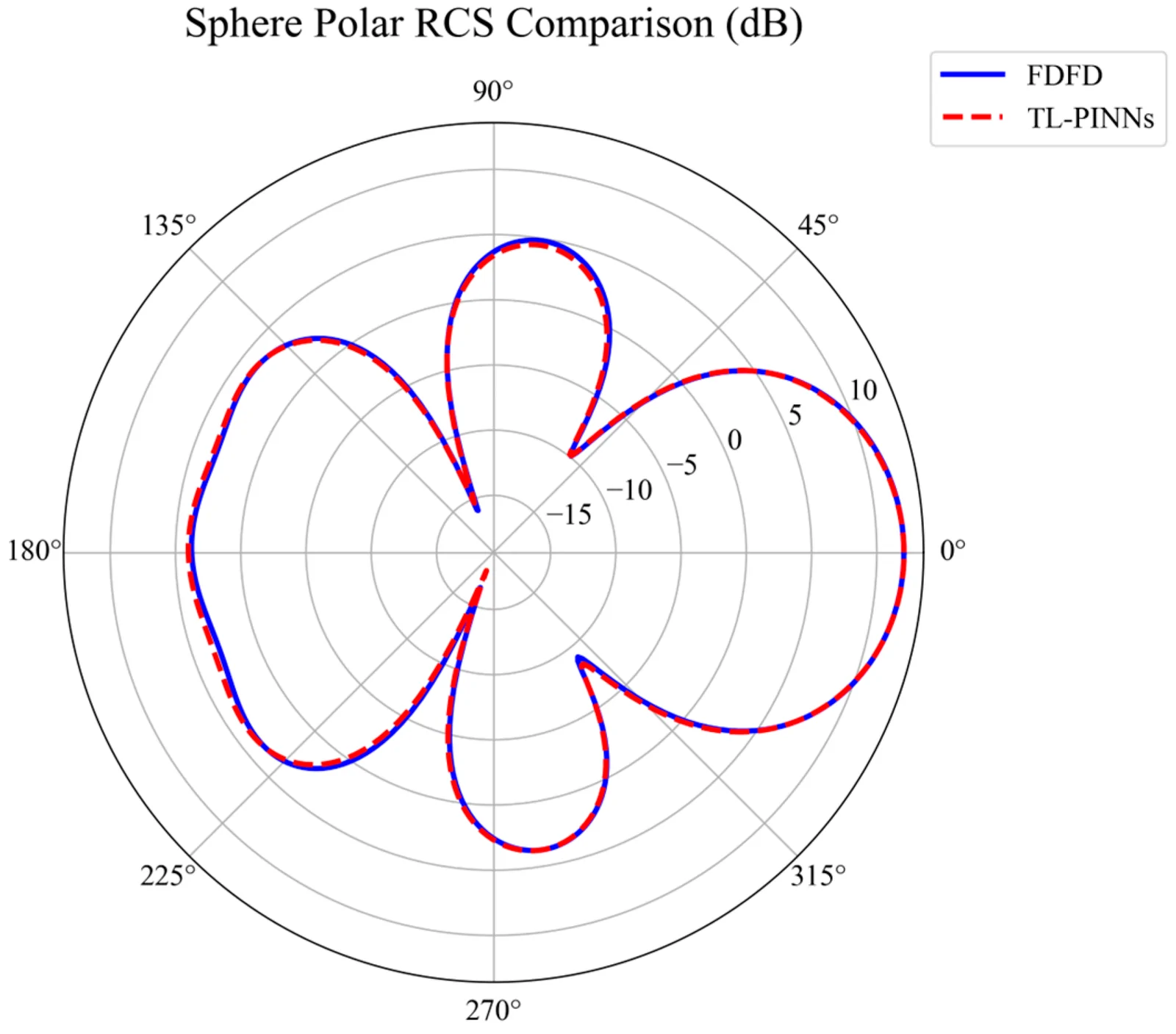
Far-field bistatic Radar Cross-Section (RCS) polar comparison for the baseline dielectric sphere. The plot demonstrates the high predictive accuracy of macroscopic optical properties by comparing the RCS calculated using the pre-trained PINN model (red dashed line) with the FDFD benchmark (blue solid line).

However, in practical micro-device design, these localized near-field errors have minimal effect on macroscopic optical properties. To quantitatively verify this, the far-field bistatic Radar Cross-Section (RCS) of the baseline sphere was computed via near-to-far-field integration. As depicted in Figure 2, the PINN prediction matches the FDFD benchmark perfectly across the entire 360° angular range. This strict alignment confirms that far-field integration functions as a spatial low-pass filter, neutralizing high-frequency interfacial fluctuations and establishing a rigorous physical baseline for subsequent transfer tasks.

Despite local errors at the focal peaks and interfaces, quantitative calculations indicate strong overall performance. For this baseline scattering target, the maximum absolute error of the PINN-predicted field is 0.728. The overall relative error is 7.71%, which meets the preset acceptable accuracy limit of below 10%. To achieve this accuracy from scratch, the baseline model requires approximately 810,000 training iterations. This complete training process takes about 97,234 s (approx. 27 h). These results validate the numerical precision of the proposed model on baseline tasks, while also highlighting the necessity of transfer learning to reduce the computational time in new scenarios.

### 3.3. Analysis of Transfer Capability

To demonstrate the transfer performance of this framework, this paper will elaborate on two dimensions of transferability: cross-target transfer and cross-incident beam transfer.

#### 3.3.1. Transfer Across Geometric Targets

To illustrate the cross-target transfer capability, this paper selects a dual-sphere system, which poses greater transfer difficulty. The narrow grooves and non-smooth boundaries formed by the fusion of the two spheres increase the complexity of the spatial structure, thereby amplifying the difficulty for PINNs to fit and reconstruct locally drastically changing physical fields. The selected model has a radius of 0.133 μm (0.25 λ) and a center offset of 0.12236 μm (0.23 λ).

**Figure 3:**
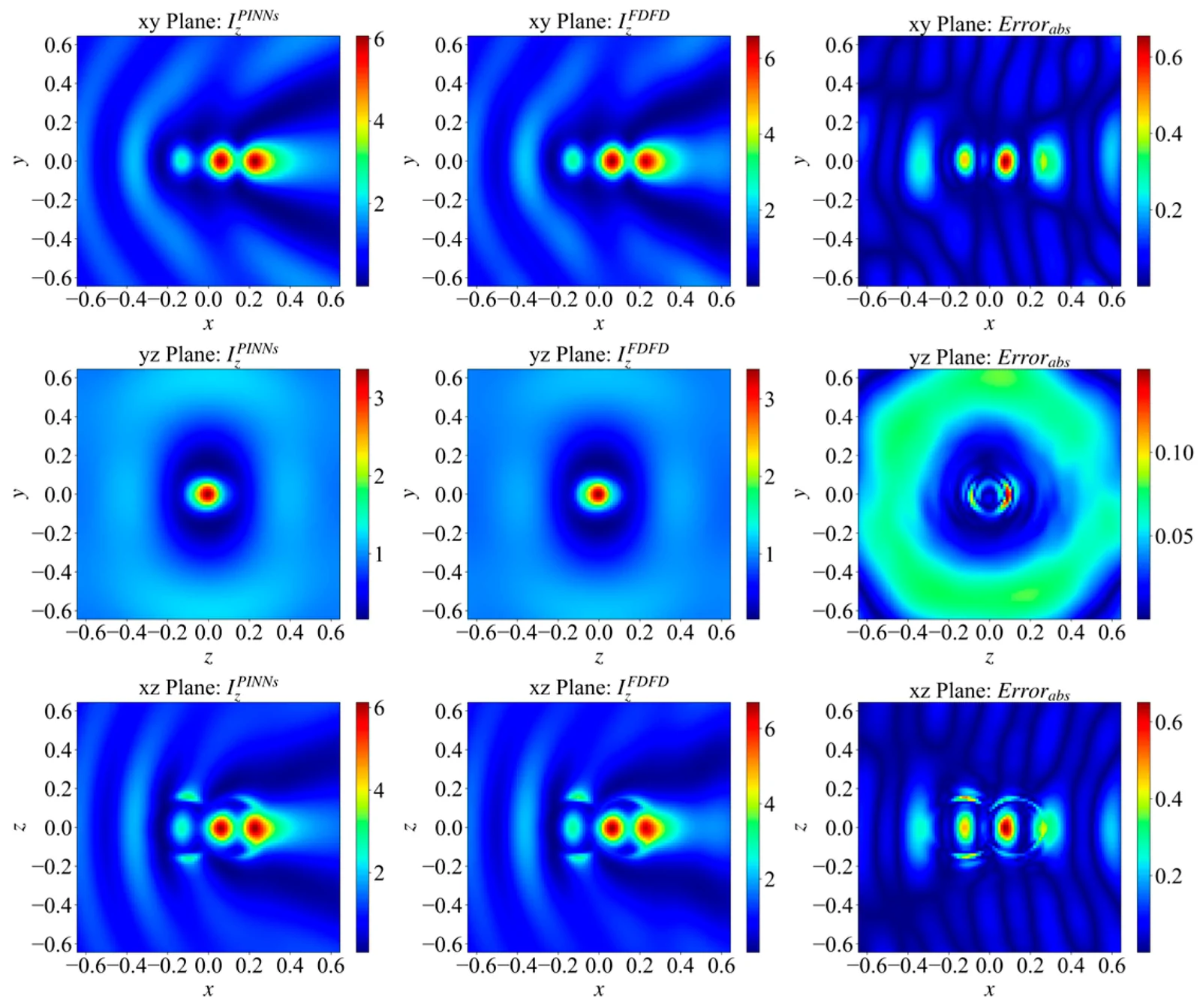
Reconstructed electric field intensity distributions on the three orthogonal principal cross-sections for the dual-sphere system. The figure demonstrates the cross-geometry transfer capability by comparing the PINN-predicted fields, the FDFD-calculated true fields, and the spatial error distributions across the xy, yz, and xz planes after transferring from the single-sphere baseline model.

The fitting results of the electric field vector intensities on the three orthogonal principal cross-sections (xy, xz, yz) after transferring the model from the original sphere to the dual-sphere system are shown in Figure 3.

By comparing the spatial distributions of the predicted field ($I_{z}^{PINNs}$) and the true calculated field ($I_{z}^{FDFD}$), it is evident that even when the scattering target is expanded from a single sphere to a dual-sphere structure, the transferred PINN model still maintains high consistency with the benchmark solution in terms of overall wavefield morphology. The network not only accurately reconstructs the strong local focal regions formed by electromagnetic waves inside the dual spheres but also commendably restores the complex interference fringes dominated by multiple scattering in the external space. Notably, observing the distribution details in the yz plane reveals that some intensity fitting deviation still exists between the predicted field and the true calculated field in the near-field coupling gap region between the two spheres.

Analysis of the spatial distribution characteristics of the absolute error (Error) reveals that the error extreme also exhibits a high degree of regional clustering: they are mainly concentrated at the physical interfaces between the dual-sphere medium and the vacuum, as well as at the high-frequency focal peaks inside the target. This phenomenon aligns with the error distribution patterns observed in the baseline geometric target (single-sphere model), further indicating that continuous neural networks possess inherent representational limitations in characterizing spatial step mutations in permittivity and in fitting high-frequency local maxima.

Quantitative evaluation results demonstrate that for this dual-sphere cross-geometry transfer task, the maximum absolute error of the predicted field is 0.652, and the overall relative error is 9.90%. This result satisfies the acceptable accuracy limit requirement of a relative error below 10%, verifying that the proposed solution framework still possesses reliable physical feature transfer and 3D high-frequency scattered field solving capabilities when facing increased geometric complexity of scatterers.

**Figure 4:**
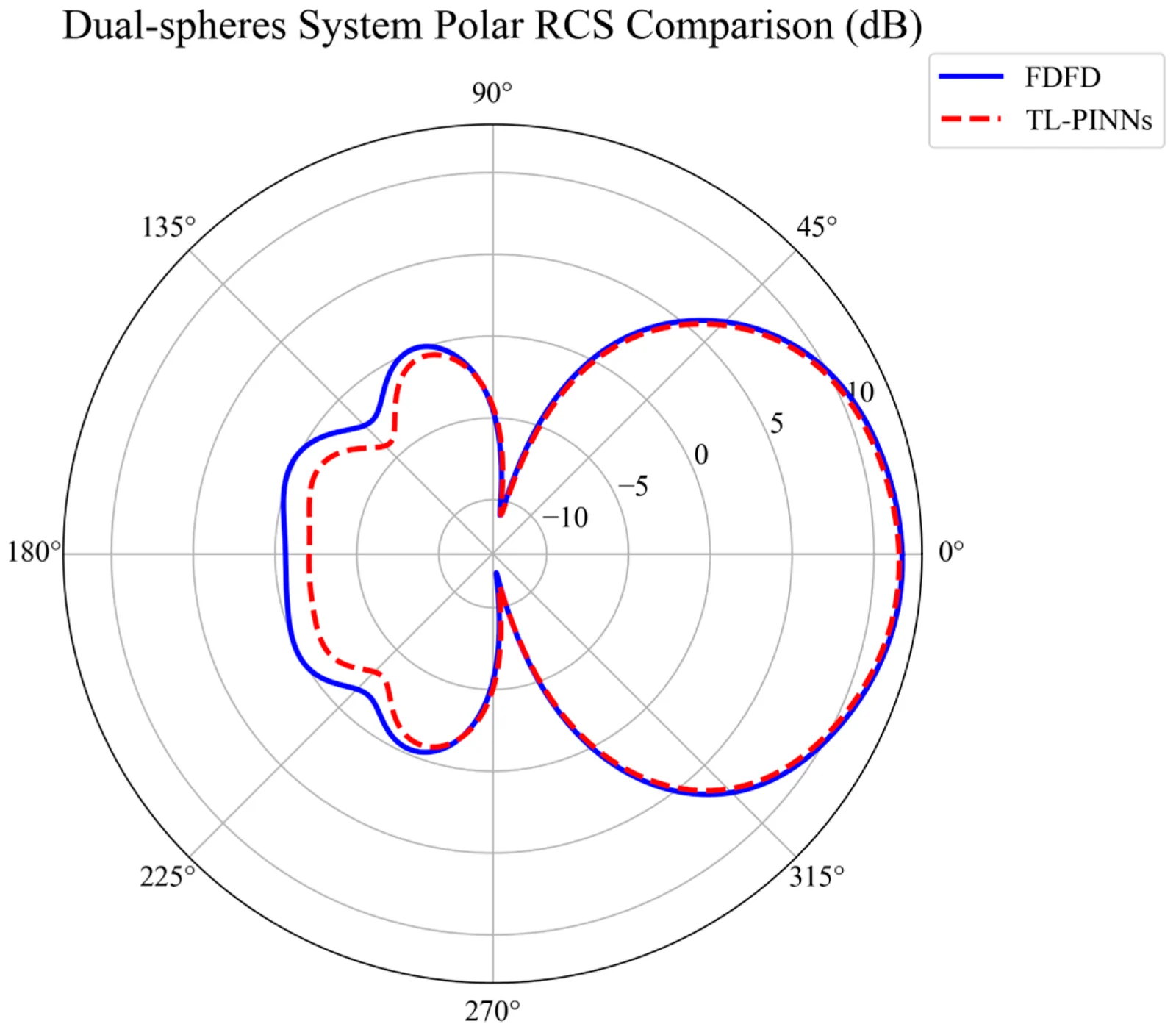
Far-field bistatic Radar Cross-Section (RCS) polar comparison for the dual-sphere system. The plot demonstrates the preservation of macroscopic scattering characteristics by comparing the RCS predicted by the transferred TL-PINN model (red dashed line) against the FDFD benchmark (blue solid line).

To evaluate the robustness of macroscopic predictions under cross-geometry transfer, the far-field RCS of the dual-sphere system was further analyzed. As illustrated in Figure 4, the transferred model exactly aligns with the FDFD benchmark in the forward-scattering hemisphere (near 0°), which dominates the total scattered energy. Although a minor magnitude deviation exists in the low-energy backscattering region (near 180°) due to its inherent sensitivity to boundary perturbations, the global macroscopic scattering pattern is accurately preserved. This validates the reliable far-field transferability of the TL-PINN framework, demonstrating its capability to maintain physical fidelity for complex device evaluation.

To further validate the cross-target transfer capability, this paper conducts transfer validation from the pre-trained baseline geometric target to several typical complex scatterers: an ellipsoid (semi-axes a=0.5λ,b=0.25λ,c=0.25λ), a hemisphere (radius $r_{h}=0.4\lambda$), a cube (side length L=0.8λ), and the aforementioned dual-sphere system (radius $r_{s}=0.25\lambda$, center offset d=0.23λ). By diversifying the spatial topologies and boundary discontinuities of the targets, the robustness of the model can be comprehensively evaluated. The following Table 1 summarizes the transfer solution results for these target geometries under different freezing strategies. These results include the computational running time measured in seconds (s). This training time represents the total duration required for the transfer fine-tuning optimization process to converge under the new geometric constraints. Additionally, the computation time listed in the table represents the actual duration required for the transferred model to load the fine-tuned weights and perform rapid forward propagation to output the full 3D scattered field. In all cases, the maximum number of transfer iterations is set to 110,000.

For the PINN architecture containing 4 hidden layers, Table 1 quantitatively compares the absolute errors, relative errors, convergence iteration steps, and the required running time (in seconds) of the model’s cross-geometry target transfer under different fine-tuning strategies (freezing 1, 2, or 3 layers).

When the “freeze 3 layers” strategy is adopted, the optimization for all target geometries hits the maximum iteration steps (110,000 times), and none of the relative errors drop below the acceptable accuracy limit of 10%. In terms of network capacity, extensively freezing deep layers restricts the model to a single layer for reconstructing entirely new, complex geometric boundary conditions. Its restricted parameter degrees of freedom struggle to support large-span spatial topological mappings.

Conversely, when the “freeze 1 layer” strategy is utilized, all geometries except the ellipsoid model successfully converge, and the iteration overhead exhibits a sharp decrease compared to the “freeze 3 layers” strategy. However, the iteration steps of this scheme remain relatively high (e.g., the hemisphere model requires 49,500 iterations. The ellipsoid model, although falling short of the standard, reaches the pre-defined maximum iteration limit (110,000 steps) with the relative error stagnating at 11.9%. Physically, freezing too few layers forces many intermediate-layer neurons—which have already extracted universal wave priors—back into the optimization process. The excessive degrees of freedom in the tunable parameters not only destroy the solidified underlying electromagnetic operator features [65], triggering severe oscillations in the loss curve, but also introduce obvious computational redundancy.

In comparison, the “freeze 2 layers” strategy demonstrates the lowest error levels and the fewest iteration steps across all cross-geometry tests. Various scatterer models achieve stable convergence, with the iteration steps for the hemisphere and cube models further compressed to 30,500 and 27,500, respectively. Moreover, the ellipsoid model, which exhibited lower precision under other strategies, successfully meets the acceptable accuracy limit at 59,000 iterations. This indicates that freezing two network layers strikes an optimal balance between physical constraints and geometric fitting: locking the shallow network (the first two layers) effectively preserves the universal Maxwell wave priors, while opening the deep network (the last two layers) provides sufficient parameter space to fit the novel geometric boundaries precisely.

Synthesizing the above data analysis reveals that in cross-geometry target transfer tasks, an appropriate layer-freezing ratio is crucial for ensuring the effective reuse of physical features. The proposed framework adopts an optimal ‘freeze 2 layers’ strategy. With this strategy, it successfully verifies the reliability of cross-topological structure solving. More importantly, the optimal ‘freeze 2 layers’ strategy drastically reduces the computational cost of predicting a new physical field. It brings the required iterations down from approximately 810,000 in the baseline model to just 27,500–59,000. Correspondingly, the running time is reduced from about 27 h to merely 1 to 2 h.

Finally, it is worth highlighting the advantage of the proposed TL-PINNs. As shown in Table 1, once model transfer is complete and the transferred weights are loaded, the network requires only 4–5 s to compute the full 3D scattered field. In contrast, a GPU-accelerated FDFD simulation achieving comparable accuracy takes approximately 10 min, representing an efficiency improvement of nearly 120 times. Since FDFD is generally more efficient than FDTD for this type of problem, the efficiency advantage of the proposed method over FDTD could be even more pronounced [7,9]. This result demonstrates the substantial potential of the proposed method for rapid electromagnetic evaluation.

**Figure 5:**
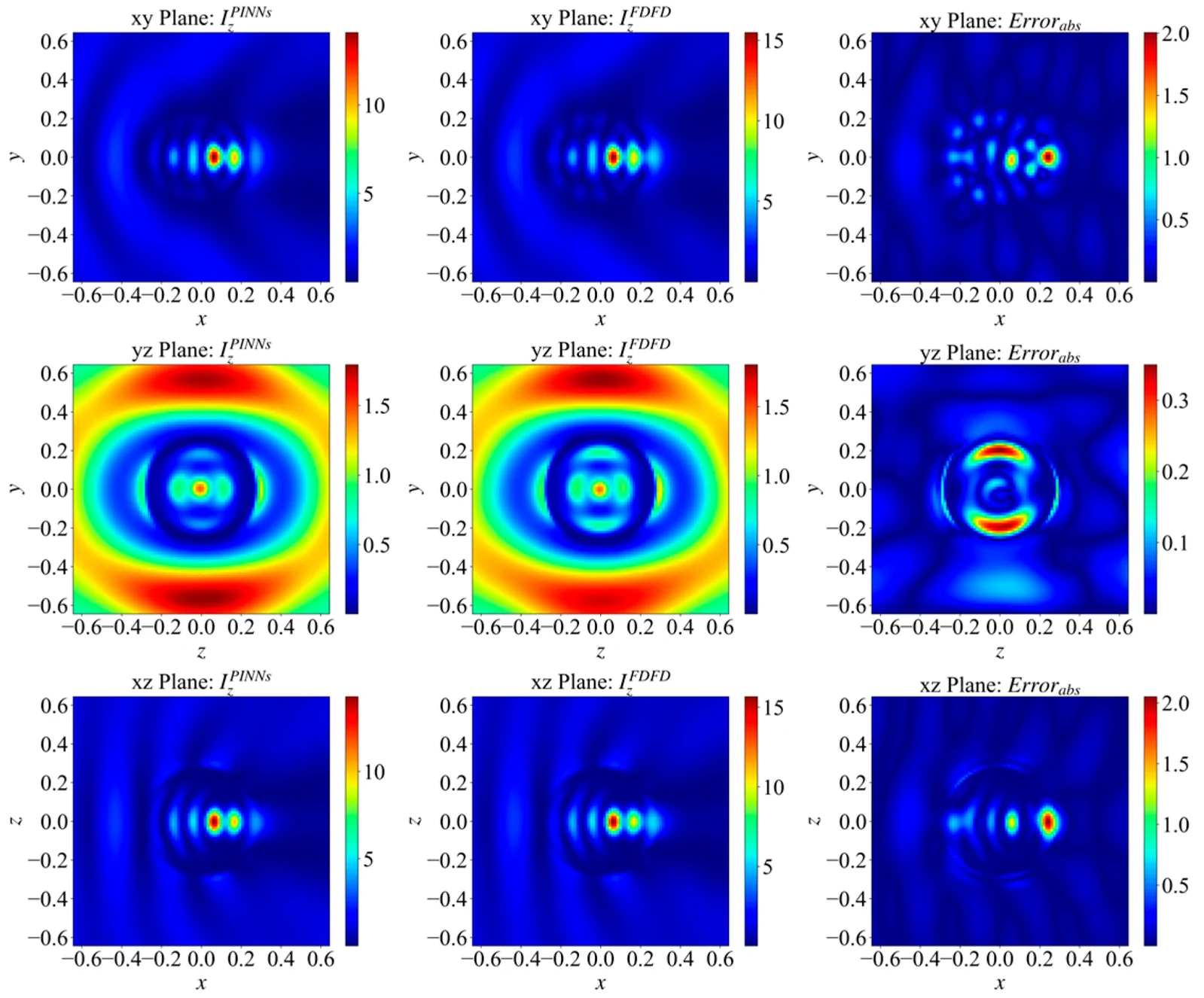
Reconstructed electric field intensity distributions on the three orthogonal principal cross-sections for the high-contrast dielectric medium. The figure demonstrates the cross-material transfer capability by comparing the transferred TL-PINN-predicted fields, the FDFD-calculated benchmark fields, and the spatial error distributions across the xy, yz, and xz planes after transferring from the baseline model to a medium with a relative permittivity of 10.0.

Beyond geometric variations, to clarify the applicability boundaries of the TL-PINN framework, Figure 5 evaluates its transferability to a high-contrast dielectric medium ($\epsilon_{r}=10.0$). Inside this medium, the compressed effective wavelength causes severe high-frequency phase oscillations. As a result, the transferred network captures the overall wavefield well, but it struggles to resolve the exact peak intensities of dense focal spots.

Using the optimal “freeze 2 layers” strategy, the transferred TL-PINN model converges after 45,500 iterations with a relative error of 12.6%. Although this marginally exceeds the 10% threshold, the global field distribution still closely matches the FDFD benchmark. This verifies that the layer-freezing strategy remains feasible and robust even under significant material variations. Such accuracy is practically acceptable for rapid structural prototyping.

However, this test reveals a clear scaling limit. Extending the method to higher-contrast materials ($\epsilon_{r}>10$) severely exacerbates the spectral bias of PINNs [63,64], which consequently limits the scaling capability of the current TL-PINN framework. Future studies will need multi-scale grid mapping or domain decomposition strategies [66] to handle the extreme spatial gradients in highly resonant systems.

**Figure 6:**
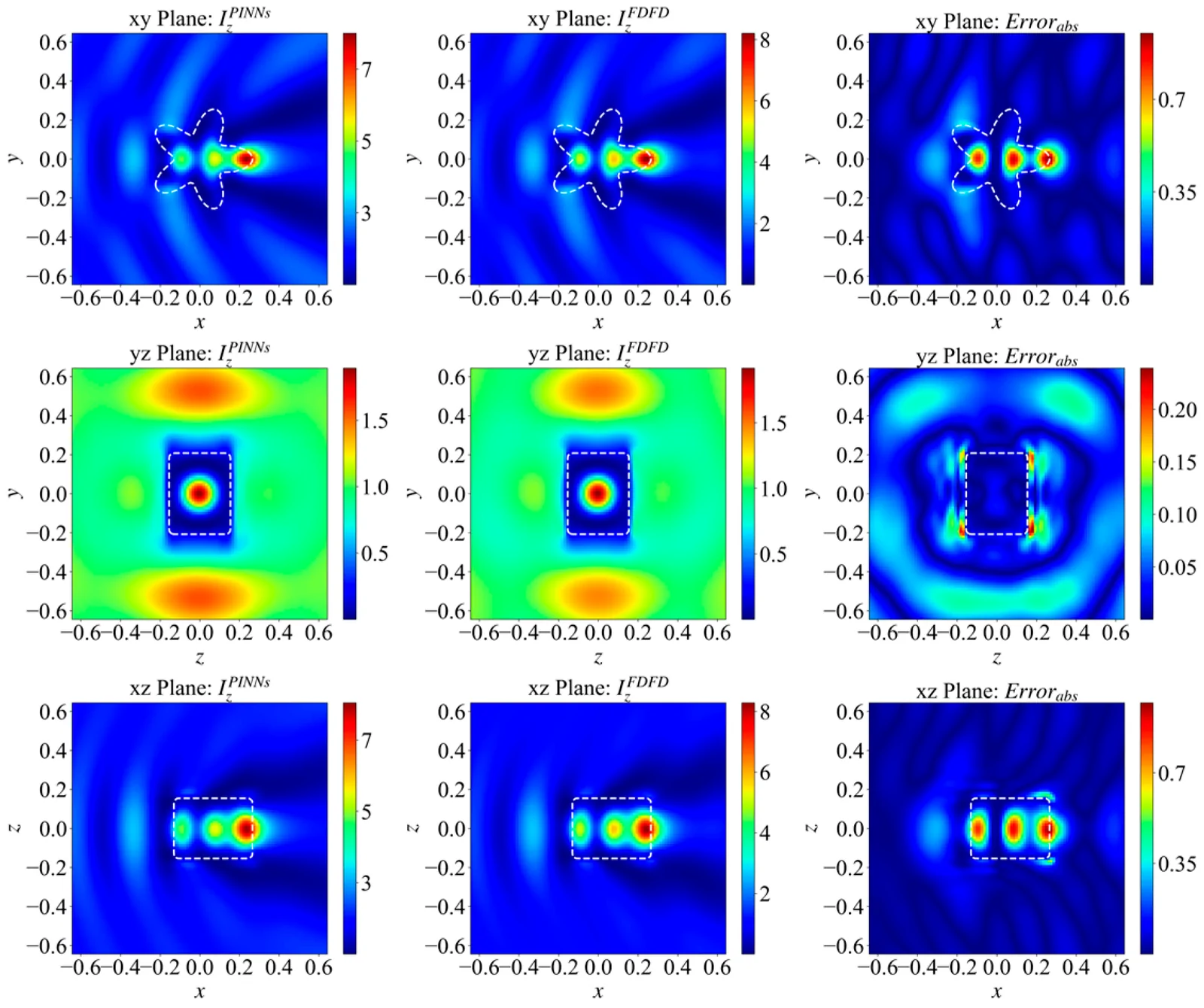
Reconstructed electric field intensity distributions on the three orthogonal principal cross-sections for a highly complex non-convex target (a cylindrical star with rounded corners). The figure demonstrates the geometric transfer limit by comparing the PINN-predicted fields, the FDFD-calculated benchmark fields, and the spatial error distributions across the xy, yz, and xz planes after transferring from the baseline single-sphere model.

To further test the transfer capability of the TL-PINN framework, we introduced a more complex target: a cylindrical star with rounded corners. The geometric parameters of this target are set as follows: the height is h=0.5λ, the outer vertex radius is $R_{outer}=0.46\lambda$, the inner concave radius is $R_{inner}=0.20\lambda$, and the edge smoothing radius is $r_{smooth}=0.04\lambda$. This geometry has non-smooth edges and complex concave-convex boundaries. These geometric features typically cause severe spatial gradient mutations. However, as shown in Figure 6, the transferred model successfully captures the global scattering patterns, local focal spots, and complex interference fringes.

Using the optimal “freeze 2 layers” strategy, the relative error for this extreme geometry is approximately 13.3%. This value slightly exceeds our preset 10% threshold. Nevertheless, the framework still accurately reconstructs the primary physical wavefield features. This proves its robustness against topological complexities that are far beyond the pre-trained baseline. Ultimately, it provides a highly valuable reference for the rapid prototyping of complex optical structures.

In summary, we tested the TL-PINN framework across various geometries. These tests ranged from basic shapes to high-contrast media and complex non-convex targets. The results clearly demonstrate the framework’s robustness and physical boundaries. We found that the ‘freeze 2 layers’ strategy is optimal. It effectively reuses universal electromagnetic wave priors. At the same time, it keeps enough parameter freedom to fit new spatial boundaries. In extreme cases, such as the cylindrical star or highly resonant systems ($\epsilon_{r}>10$ or electrical size >5λ), the relative error slightly exceeds 10%. However, the framework still accurately reconstructs the global scattering patterns and local field characteristics. Ultimately, these findings reveal the scaling limits of continuous neural networks at high frequencies. Despite this, they fully confirm the framework’s high reliability and computational efficiency for 3D cross-target transfer tasks.

#### 3.3.2. Transfer Across Complex Incident Beams

In addition to geometric morphological changes, practical optical systems are often accompanied by illumination from complex modulated beams. Therefore, while keeping the scattering geometric target (dielectric sphere) constant, this paper switches the plane wave excitation source in the pre-trained model to other typical beams propagating along the x-direction and polarized in the z-direction. Introducing the transverse (yz plane) cylindrical coordinate parameter $\rho =\sqrt{y^{2}+z^{2}}$ and the azimuthal angle ϕ=arctan(z/y), the analytical expressions for each incident field $E_{inc}$ are defined as follows:

Gaussian beam [67]:

To strictly enforce the zero-divergence condition ($\nabla \cdot E_{inc}=0$) under the paraxial approximation, the incident field is constructed as a linearly polarized vector Gaussian beam. Specifically, $E_{inc}=E_{inc,x}x+E_{inc,z}z$ (with the y-polarization component strictly defined as $E_{inc,y}=0$ across the entire domain, as cross-polarization is a negligible second-order effect in this first-order approximation model). The primary transverse component $E_{inc,z}$ and the required first-order longitudinal component $E_{inc,x}$ are respectively given by:

(25) $$E_{inc,z}=-ik\Psi_{0}e^{-ikx},$$

(26) $$E_{inc,x}=-\frac{2Qz}{l}E_{inc,z},$$

where k is the wavenumber, $l=kw_{0}^{2}$ is the characteristic diffraction length ($w_{0}$ being the beam waist parameter), and z is the transverse coordinate along the polarization direction. The fundamental scalar mode solution $\Psi_{0}$ is defined as $\Psi_{0}=exp[-i(P+Q\rho^{2})]$. The complex parameters Q and P are given by Q=1/(i+2ζ) and iP=−ln(iQ), with ζ=x/L being the dimensionless propagation variable along the optical axis.

Bessel beam [68,69]:

(27) $$E_{inc}=E_{0}J_{n}(k_{t}\rho )exp(in\phi )exp(-ik_{x}x)z,$$

where n is the beam order. $J_{n}$ is the n-th order Bessel function of the first kind. $k_{t}=k\sin\theta$ and $k_{x}=k\cos\theta$ are the transverse and longitudinal wavevectors, respectively (θ is the cone angle). exp(inϕ) is the azimuthal phase factor that imparts orbital angular momentum to the beam, and z is the polarization direction.

Specifically, when n = 1, the beam is a first-order Bessel beam. As seen from Equation (27), the first-order Bessel beam carries orbital angular momentum [70], and its most prominent physical feature is the presence of a phase singularity on the central propagation axis (ρ=0) [71]. Because $J_{1}(0)=0$, it manifests as zero field intensity at the center (i.e., the central dark core phenomenon), and its cross-sectional phase exhibits a helical distribution.

Due to the highly complex phase and spatial structure of this beam, this paper selects a first-order Bessel beam with a cone angle of θ=10° for illustration.

**Figure 7:**
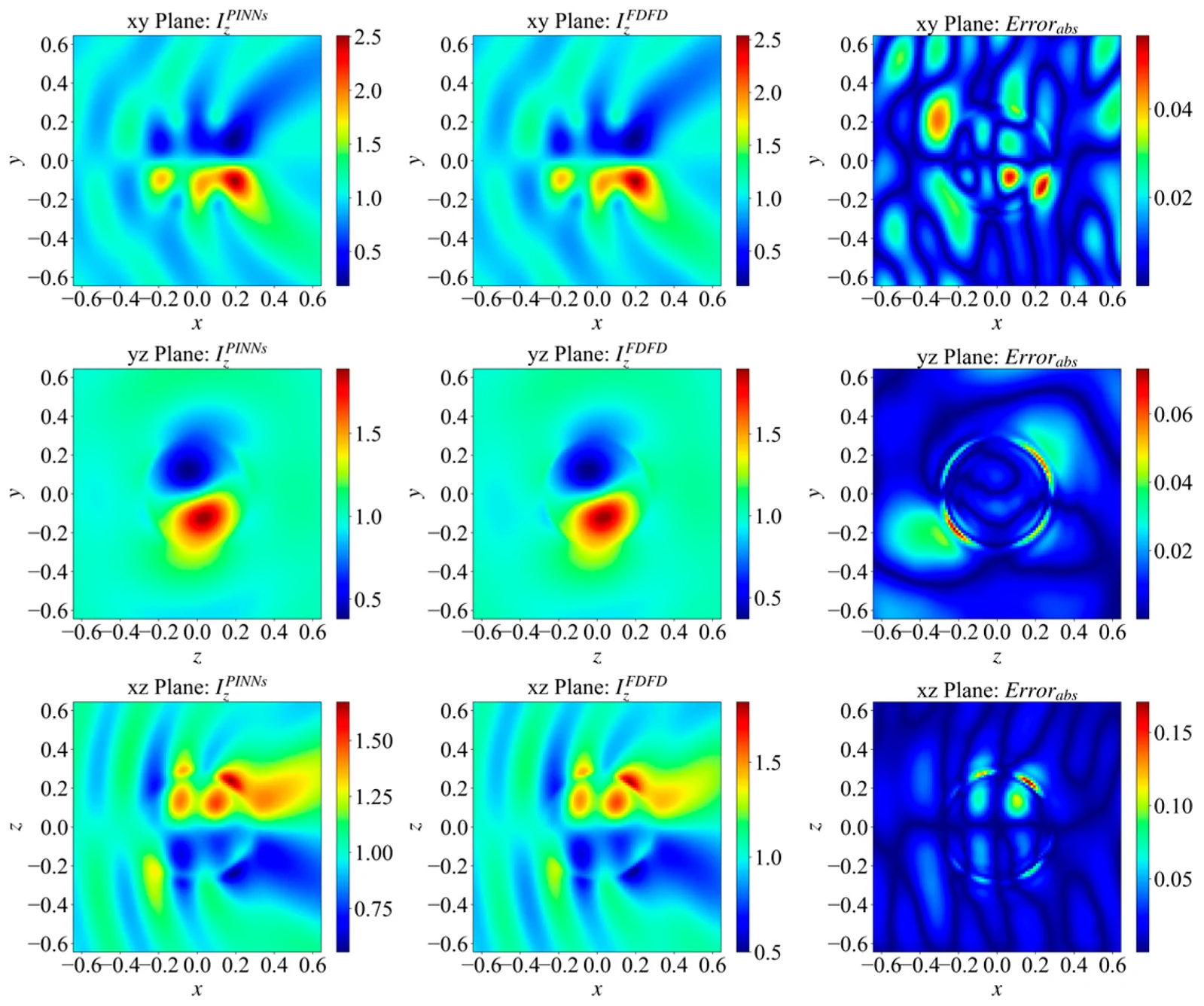
Complex 3D physical field distributions of the dielectric sphere under the illumination of a first-order Bessel beam. The subplots illustrate the cross-beam transfer performance by comparing the PINN-predicted fields, the FDFD benchmark fields, and the relative error distributions, accurately capturing the central dark core phenomenon and phase characteristics of the Bessel beam.

According to Figure 7, comparing the predicted field ($I_{z}^{PINNs}$) and the true calculated field ($I_{z}^{FDFD}$), it can be seen that the model accurately reconstructs the complex 3D physical field distribution following the interaction between the first-order Bessel beam and the dielectric sphere. Observing the xy and xz cross-sections containing the propagation axis reveals that PINNs precisely capture the core feature of the first-order Bessel beam: the field intensity on the central propagation axis is approximately zero (exhibiting a typical central dark core phenomenon), while highly antisymmetric field intensity extrema distributions appear on both sides of the central axis.

Qualitative observation of the spatial distribution characteristics of the error demonstrates that despite significant increases in the phase structure and spatial complexity of the incident beam, the aggregation pattern of the predicted field error remains consistent with that of the cross-geometry topology transfer task. The absolute errors are still predominantly concentrated at the physical interfaces of the dielectric sphere.

Calculation results show that the average absolute error of this cross-beam transfer model is 0.0206, and the overall relative error is 5.37%. This precision metric satisfies the acceptable accuracy limit of less than 10%, fully validating that the proposed solution framework also possesses efficient and robust physical feature transfer solving capabilities when faced with increasingly complex incident beam conditions.

The following table summarizes the transfer solution results for various incident waves under different freezing strategies, with the maximum transfer iterations set to 110,000.

To further verify the transfer capability, this paper conducts transfer validation on the pre-trained single-sphere model using a Gaussian beam (waist radius ${\mathrm{ssw}}_{0}=0.5\lambda$), a first-order Bessel beam (cone angle θ=10°), and a zero-order Bessel beam (cone angle θ=10°). Table 2 summarizes the transfer solution results and their corresponding training times (in seconds) under different freezing strategies, with the maximum number of iterations set to 110,000. Additionally, the computation time listed in the table represents the actual duration required for the transferred model to load the fine-tuned weights and perform rapid forward propagation to output the full 3D scattered field.

Based on the data for different freezing depths shown in Table 2, we can deduce the following rules:

The experimental results present convergence patterns highly consistent with the cross-geometry transfer tasks. When employing the “freeze 3 layers” and “freeze 1 layer” strategies, the model encounters either under-fitting (relative error > 90%) or computational redundancy (e.g., the first-order Bessel beam consumes 85,000 iterations). This phenomenon further corroborates the previous physical mechanism analysis: excessive parameter freezing in deep networks deprives the model of the degrees of freedom required to adapt to completely new phase distributions and energy gradients, while excessive opening of shallow networks unnecessarily destroys the already solidified universal wave operators.

In comparison, the “freeze 2 layers” strategy continues to exhibit outstanding robustness in cross-beam tests, compressing the convergence iterations for the Gaussian beam and the first-order Bessel beam to 15,000 and 26,000, respectively. In terms of time cost, these predictions are achieved in only about 30 to 50 min. Notably, in the transfer task to the zero-order 10° Bessel beam, the model requires a mere 270 iterations (taking 1 min) to reduce the relative error to 7.24%. This extreme convergence efficiency is attributed to the physical similarity between the zero-order Bessel beam and the plane wave in the paraxial region and directly proves the profound advantage of the proposed strategy: by locking the foundational physical perception network, the model can unleash immense rapid adaptation potential when faced with new excitations that share similar fluctuation features with the source scenario.

In summary, from the plane wave of the baseline geometric target to complex waveforms, this paper has verified the feasibility of cross-incident beam transfer. The “freeze 2 layers” strategy is once again proven optimal, and the iteration counts of less than 30,000 further enhance iteration efficiency.

Consistent with the geometric transfer scenario in Section 3.3.1, the computation time for reconstructing the full 3D scattering field under these complex incident beams remains highly efficient after loading the transfer weights, confirming the framework’s stable computational efficiency across different transfer tasks.

Based on the transfer experiment results of cross-geometry targets and cross-beam conditions, it can be concluded that the TL-PINN solution framework proposed in this paper demonstrates highly reliable feature transfer capabilities. Even though the pre-training in the source scenario only extracts the wave priors under the conditions of a baseline geometry and a plane wave, when faced with novel scenarios involving changes in scatterer morphology or reconstruction of the incident field, the model can still stably converge to the accuracy level of the traditional FDFD method with significantly reduced iteration overhead and running time. This fully validates the layer-freezing strategy of “locking the shallow physical perception network and adjusting the deep boundary mapping network.” This mechanism avoids the prohibitive computational costs associated with spatial geometric changes and demonstrates robust cross-scenario reusability for complex physical excitation sources.

#### 3.3.3. Correlation Analysis Between Physical Parameters and Error Distribution

A counter-intuitive phenomenon is observed in the aforementioned transfer experiments: transferring to more complex physical scenarios does not always decrease prediction accuracy. Instead, the relative errors of some models fall below that of the pre-trained baseline (baseline relative error of 7.71%). Specifically, when transferring to highly localized beams using the “freeze 2 layers” strategy, the relative errors of the first order and zero-order Bessel beams drop further to 5.37% and 7.24%, respectively. Meanwhile, in cross-geometry target transfer, the relative errors of the hemisphere and cube models, which contain discontinuous cross-sections and sharp edges, are also as low as 7.59% and 7.56%, respectively.

In traditional computational electromagnetics, the complexification of the excitation source phase or the introduction of sharp boundaries in target geometries typically increases the difficulty of numerical solutions. This distinct characteristic exhibited by the proposed PINN framework is primarily related to its underlying optimization logic based on the minimization of the partial differential equation loss [57,72]. This can be preliminarily explored from the following two aspects:

First, the spatial distribution characteristics of the incident wave energy affect the global fitting difficulty of the PDE loss. The electromagnetic energy under plane wave excitation is uniformly distributed within the computational domain, forcing the neural network to approximate the PDE loss terms relatively evenly across the entire domain. In contrast, the energy of Bessel beams is mainly concentrated in the principal axis region, with the edge field intensity naturally decaying. This localized distribution of energy relatively concentrates the numerical contributions of the high physical response regions in the total loss function, objectively reducing the network’s fitting burden for the low-intensity edge regions. Consequently, the model can focus its limited parameter degrees of freedom on solving the PDE constraints in the core regions, which may facilitate achieving a lower total validation error in the local core area.

Second, the geometric discontinuity of the targets alters the gradient characteristics of the loss landscape. The baseline sphere model possesses high smoothness and symmetry, and its corresponding total loss function may contain numerous flat regions in the multi-dimensional parameter space. In contrast, targets containing cross-sections or sharp edges, such as hemispheres or cubes, introduce significant spatial partial derivative variations at the physical interfaces. During backpropagation, local PDE loss mutations induced by geometric discontinuities provide clearer gradient guidance for weight updates. This helps the two-stage optimizer (particularly L-BFGS) bypass flat loss regions and prevent optimization stagnation.

In summary, the localization of field intensity and non-smooth geometric features physically increases the complexity of the scenario, but at the optimization level of PINNs, they may provide more favorable convergence conditions for the descent of its loss function. This indicates that the solution accuracy of PINNs is determined not only by the network capacity but is also influenced by the effect of physical constraints on the gradient distribution along the optimization path.

## 4. Conclusions

This paper proposes a transferable solution method that integrates a discretized difference network with Physics-Informed Neural Networks (PINNs). It aims to achieve rapid prediction of 3D complex electromagnetic scattered fields. By directly embedding finite-difference Maxwell operators into the neural network’s loss function and applying a layer-freezing strategy, the constructed TL-PINN framework establishes a strong physical constraint mechanism.

Numerical simulation results demonstrate that the model achieves rapid convergence in novel geometric scenarios within merely 30,000 to 50,000 iterations. This effectively avoids the high computational cost of full-network retraining. For the four-layer neural network architecture used in this study, the ‘freeze 2 layers’ strategy is validated as the optimal approach for cross-scenario fine-tuning. Under this strategy, the framework successfully transfers to various complex geometries. Even for complex non-convex topologies (e.g., cylindrical stars) and high-contrast media ($\epsilon_{r}=10$), the model accurately reconstructs global scattering patterns. Scaling to strongly resonant systems (e.g., $\epsilon_{r}>10$ or electrical sizes >5λ) causes the relative error to exceed the 10% threshold slightly. However, the framework still effectively preserves the core physical field characteristics. These findings clearly define the scaling boundaries of continuous neural approximators in high-frequency environments.

Beyond cross-geometry transfer, the framework demonstrates robust transfer capabilities across complex modulated incident beams. The model accurately reconstructs intricate 3D wave phenomena. This includes the phase singularities and central dark cores inherent in Gaussian and Bessel beams. Under these typical target and beam excitations, the relative error of the predicted fields is stably controlled within 10%. This aligns perfectly with traditional FDFD benchmark simulations.

Furthermore, we deeply explored the correlation between physical parameters and the spatial distribution of errors. Our analysis reveals a counterintuitive phenomenon: highly localized energy distributions (e.g., Bessel beams) and sharp geometric discontinuities actually facilitate the optimization process. They concentrate the PDE loss and provide clearer gradient guidance. This ultimately helps the optimizer achieve convergence much more efficiently.

In summary, the proposed method provides high-precision, low-overhead technical support for cutting-edge applications, such as the inverse design of metasurfaces [25] and the real-time radar cross-section (RCS) analysis of complex targets. Future work will focus on rigorous comparative studies between discrete physical constraints and emerging neural operators (such as FNO and DeepONet [73]). This will systematically evaluate their training complexity, memory consumption, and grid resolution sensitivity when handling electrically large problems.

## Figures and Tables

**Table 1:** Transfer Solution Results for Target Geometries Under Different Freezing Strategies.

Target Model	Target Dimensions	Freezing Strategy	Absolute Error	Relative Error	Number of Iterations	Training Time	Computation Time
Ellipsoid	a = 0.5λ b = 0.25λ c = 0.25λ	1 layer	0.596	11.9%	110,000	11,538 s	4.45 s
		2 layers	0.445	8.92%	59,000	5792 s	4.63 s
		3 layers	0.678	13.5%	110,000	10,892 s	4.39 s
Hemisphere	$r_{h}=0.4\lambda$	1 layer	0.237	7.59%	49,500	4860 s	5.20 s
		2 layers	0.281	8.94%	30,500	2998 s	5.17 s
		3 layers	0.361	11.5%	110,000	11,745 s	5.27 s
Cube	L=0.8λ	1 layer	1.11	8.88%	30,500	2965 s	5.24 s
		2 layers	0.949	7.56%	27,500	2594 s	5.18 s
		3 layers	1.41	11.2%	110,000	10,491 s	5.11 s
Dual-sphere system	$r_{s}=0.25\lambda$ d=0.23λ	1 layer	0.599	9.12%	50,500	5061 s	4.98 s
		2 layers	0.652	9.90%	46,500	4586 s	5.07 s
		3 layers	0.945	14.2%	110,000	11,386 s	5.14 s

**Table 2:** Transfer Solution Results for Incident Waves Under Different Freezing Strategies.

Target Model	Target Dimensions	Freezing Strategy	Absolute Error	Relative Error	Number of Iterations	Training Time	Computation Time
Gaussian beam	$w_{0}=0.5\lambda$	1 layer	0.777	9.67%	21,500	2209 s	7.14 s
		2 layers	0.683	8.51%	16,500	1473 s	7.26 s
		3 layers	7.33	92.2%	110,000	12,807 s	7.35 s
Bessel beam	First-order θ=10°	1 layer	0.0429	9.23%	85,000	8345 s	6.77 s
		2 layers	0.0206	5.37%	26,000	2553 s	6.51 s
		3 layers	0.369	96.1%	110,000	11,654 s	6.62 s
	Zero-order θ=10°	1 layer	1.54	9.98%	41,000	4023 s	7.34 s
		2 layers	1.13	7.24%	270	67 s	7.26 s
		3 layers	14.1	91.3%	110,000	11,038 s	7.29 s

## Data Availability

The data presented in this study are available on request from the corresponding author.

## Abbreviations

PINNs	Physics-Informed Neural Networks
TL-PINN	Transfer Learning Physics-Informed Neural Networks
3D	Three-dimensional
FDFD	Finite-difference frequency-domain
FDTD	Finite-difference time-domain
FEM	Finite element method
PML	Perfectly matched layer
MSE	Mean squared error
PDE	Partial differential equation
MLP	Fully connected feedforward neural network
SDF	Signed Distance Function
RCS	Radar cross-section
